# Response-Modality-Specific Encoding of Human Choices in Upper Beta Band Oscillations during Vibrotactile Comparisons

**DOI:** 10.3389/fnhum.2017.00118

**Published:** 2017-03-15

**Authors:** Jan Herding, Simon Ludwig, Felix Blankenburg

**Affiliations:** ^1^Neurocomputation and Neuroimaging Unit, Department of Education and Psychology, Freie Universität Berlin,Berlin, Germany; ^2^Bernstein Center for Computational Neuroscience Berlin,Berlin, Germany

**Keywords:** beta band, EEG, decision making, vibrotactile, saccade

## Abstract

Perceptual decisions based on the comparison of two vibrotactile frequencies have been extensively studied in non-human primates. Recently, we obtained corresponding findings from human oscillatory electroencephalography (EEG) activity in the form of choice-selective modulations of upper beta band amplitude in medial premotor areas. However, the research in non-human primates as well as its human counterpart was so far limited to decisions reported by button presses. Thus, here we investigated whether the observed human beta band modulation is specific to the response modality. We recorded EEG activity from participants who compared two sequentially presented vibrotactile frequencies (f1 and f2), and decided whether f2 > f1 or f2 < f1, by performing a horizontal saccade to either side of a computer screen. Contrasting time-frequency transformed EEG data between both choices revealed that upper beta band amplitude (∼24–32 Hz) was modulated by participants’ choices before actual responses were given. In particular, “f2 > f1” choices were always associated with higher beta band amplitude than “f2 < f1” choices, irrespective of whether the choice was correct or not, and independent of the specific association between saccade direction and choice. The observed pattern of beta band modulation was virtually identical to our previous results when participants responded with button presses. In line with an intentional framework of decision making, the most likely sources of the beta band modulation were now, however, located in lateral as compared to medial premotor areas including the frontal eye fields. Hence, we could show that the choice-selective modulation of upper beta band amplitude is on the one hand consistent across different response modalities (i.e., same modulation pattern in similar frequency band), and on the other hand effector specific (i.e., modulation originating from areas involved in planning and executing saccades).

## Introduction

One of the most complete pictures of neural processes involved in perceptual decision making emerges from the seminal work that has been done in the somatosensory domain over the last years (see Romo and de Lafuente, 2013 for a comprehensive review). Romo and colleagues scrutinized neuronal activity in non-human primates during all stages of a vibrotactile two-alternative forced choice (2AFC) task. In this task, monkeys had to compare two frequencies (f1 and f2) that were presented one after another, separated by a short working memory (WM) period. Decisions about whether f2 > f1 or f2 < f1 had to be reported via button press after the presentation of f2. Electrophysiological recordings revealed that firing rates in somatosensory cortices (primary and secondary; SI and SII) scaled with the stimulus frequency during presentation (Hernández et al., 2000), whereas prefrontal cortex (PFC) firing rates mirrored f1 (i.e., the frequency) during the WM period (Romo et al., 1999; see also Barak et al., 2010). Most importantly, firing rates in medial and ventral premotor cortex (mPMC and vPMC) encoded the upcoming choices of the monkeys for correct and incorrect decisions (Hernández et al., 2002; Romo et al., 2004).

More recently, Haegens et al. (2011) showed that the monkeys’ choices in the vibrotactile 2AFC task were also reflected by amplitude modulations of beta band oscillations (∼18–26 Hz) in premotor local field potentials (LFPs). Applying the same task in a human electroencephalography (EEG) study, we found that this result also translates into beta band oscillations recorded at the scalp (Herding et al., 2016). In particular, the amplitude of upper beta band oscillations (∼20–30 Hz), most likely originating from medial premotor areas, was higher when participants chose “f2 > f1” as compared to “f2 < f1,” for correct and for incorrect decisions. These findings match the results of Haegens et al. (2011), and hence, nicely complement the body of work by Romo and colleagues in non-human primates (see above).

According to the notion of an intentional framework of decision making, neural correlates of decisions should be found in brain areas that are involved in the planning and execution of the ensuing motor response (e.g., Cisek, 2007; Shadlen et al., 2008; Cisek and Kalaska, 2010). The work in non-human primates, as well as our recent study, required choices to be reported by a button press. Thus, observing choice-specific neural activity in premotor areas, for planning and informing an ensuing button press, is in line with an intentional framework of decision making. The importance of the intentional framework has been fostered in particular by the extensive body of work compiled by Shadlen and co-workers (reviewed in Gold and Shadlen, 2007). In the visual domain, perceptual decisions that are expressed by saccades, involve those brain areas that are responsible for saccade planning/execution, i.e., lateral intraparietal area (LIP; e.g., Shadlen and Newsome, 1996), frontal eye fields (FEF; e.g., Kim and Shadlen, 1999), and superior colliculus (SC; e.g., Ratcliff et al., 2003).

Taken together, each of the two major lines of research on perceptual decision making in non-human primates (cf. Gold and Shadlen, 2007; Romo and de Lafuente, 2013) appears to converge towards the notion of an intentional framework of decision making. However, the findings from both approaches (vibrotactile button press decisions and visual saccade decisions) have not yet been linked, and thus it is still unclear whether the respective results are directly transferable. In the present study, we aimed to bridge the gap between these two lines of research. We used the vibrotactile 2AFC task typically utilized by Romo and colleagues combined with saccade responses as applied in most of the work by Shadlen and colleagues. In particular, we investigated whether the choice-specific beta band modulation that we observed in our recent study (Herding et al., 2016) would still be present when participants were asked to respond with saccades instead of button presses. If so, can such a modulation be attributed to a brain area that is involved in the planning and execution of saccades as predicted by an intentional framework of decision making? To address these questions, we recorded EEG data of human participants during the vibrotactile 2AFC task, where choices were indicated by horizontal saccades. We contrasted the time-frequency (TF) transformed response-locked EEG data between both alternative choices (“f2 > f1” vs. “f2 < f1”) to reveal oscillatory signatures of decision making before responses were given. In line with the results from our previous study with button press responses (Herding et al., 2016), we found again a choice-selective modulation of upper beta band oscillations (∼24–32 Hz) in frontal electrodes. However, source localization of the choice signal suggested more lateral premotor areas as compared to medial premotor areas for the button press responses, importantly, including FEF.

## Materials and Methods

### Participants

Twenty four healthy, right-handed volunteers (20–36 years; nine males) participated in the experiment after giving written informed consent in accordance with the Declaration of Helsinki. The study was approved by the local ethics committee at the Freie Universität Berlin. Two participants (both female) were excluded from the analysis due to near chance-level behavioral performance (<60% correct answers), resulting in 22 data sets for further analysis.

### Stimuli and Behavioral Task

**FIGURE 1:**
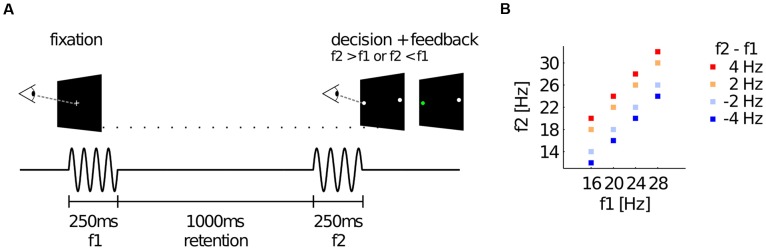
**Experimental paradigm and stimulus set. (A)** Illustration of a single trial. Participants were presented with two vibrotactile stimuli (with frequencies f1 and f2) at the left index finger, while holding central fixation until the offset of the second stimulus. Afterwards, they decided whether f2 > f1 or f2 < f1 by means of a horizontal saccade. Online feedback was provided immediately after the decision via color change of the selected dot (green for correct, red for incorrect trials). **(B)** The set of all possible frequency combinations of f1 and f2 that were applied in this study. Color-coded squares each indicate one stimulus pair with according f1 and f2 values.

Supra-threshold vibrotactile stimuli with constant peak amplitude were applied to the left index finger using a piezoelectric Braille stimulator (QuaeroSys, Schotten, Germany). The stimuli consisted of amplitude-modulated sinusoids with a fixed carrier frequency of 137 Hz. The amplitude-modulation of this carrier signal with frequencies 12–32 Hz created the sensation of tactile ‘flutter’ (see Talbot et al., 1968; Romo and Salinas, 2003), while the spectrum of the physical driving signal was limited to frequencies above 100 Hz (e.g., Tobimatsu et al., 1999). Thus, the risk of physical artifacts in the EEG analysis range of interest (<100 Hz) was minimized. The sound of the stimulator was masked by white noise of ∼80 dB that was played throughout the experiment (e.g., Spitzer et al., 2010; Spitzer and Blankenburg, 2011). Participants were comfortably seated ∼60 cm in front of a TFT monitor. A fixation cross was displayed at the center of the screen to minimize eye movements. On each trial, two flutter stimuli were successively presented for 250 ms each (with frequencies f1 and f2), interleaved by a retention interval of 1000 ms (see **Figure 1A**). The values of f1 were randomly drawn from 16, 20, 24, or 28 Hz, whereas f2 differed from f1 by ±2 or 4 Hz (**Figure 1B**). After presentation of the second stimulus the central fixation cross vanished and two target dots (diameter of ∼0.5° visual angle) appeared on the left and on the right side of the screen (∼12° visual angle off-center). Participants indicated whether f2 > f1 or f2 < f1 by making a saccade to the right or to the left target, respectively. Importantly, the response assignment of saccade directions was reversed for half of the participants, such that the mapping of choices onto specific saccades (which might have been associated with specific motor preparatory signals) was fully counterbalanced across participants. Responses were registered as soon as participants fixated one of the targets for 200 ms. According choices were evaluated online to provide immediate (with a latency of 20 ms) performance feedback by changing the color of the selected target dot for 200 ms (green for correct, red for incorrect choices). After the feedback, the central fixation cross reappeared and replaced the target dots to indicate the beginning of a new trial. Participants had to fixate the central cross to start the new trial. After a variable time interval (1500–2000 ms) a new stimulus pair was presented. Participants completed seven blocks of 160 f1-vs-f2 comparisons (each block lasted ∼15 min including eye-tracker calibration), for a total of 1120 trials. Before the experiment started, participants performed ∼50 practice trials.

### Eye-Tracking

A Tobii T60 eye-tracker was used to record participants’ eye movements during each trial (binocular sampling at 60 Hz). The T60 is integrated into a 17′′ TFT monitor, and is able to track participants that are comfortably seated in front of the monitor (i.e., no chin rest required). Online evaluation of the participants’ gaze directions was implemented with custom code using the Tobii toolbox for MATLAB. Thus, we could check whether participants kept the gaze on the central fixation cross during each trial (with tolerance of ∼3° visual angle), and displayed a warning message if this was not the case (“Please keep fixation throughout the trial”). Additionally, we could read out participants’ choices (200 ms fixation on target dot with tolerance of ∼3° visual angle) and provide performance feedback online. To maintain a high tracking accuracy, the eye-tracker was calibrated before the beginning of each block using a standard 5-dot calibration procedure.

### Behavioral Analysis Using Bayesian Modeling

We estimated subjectively perceived frequency differences (SPFDs) based on the observation that participants do not compare f2 with the physical value of f1 (cf. Hellström, 1985, 2003), but rather with a value slightly shifted toward the mean of all presented stimulus frequencies (cf. Preuschhof et al., 2010; Ashourian and Loewenstein, 2011; Karim et al., 2012; Sanchez, 2014). Using the framework of Bayesian inference, we introduced this shifted version of f1, which we call f1′, as the expected value of the posterior distribution of f1 when using a Gaussian prior centered over all presented frequencies. Three free parameters (the variance of the likelihood distribution of f1, the variance of the prior distribution, and an overall response bias) were estimated in this model based on each participant’s choices (further details in Herding et al., 2016). The SPFDs are then defined as the differences f2–f1′ for each stimulus pair. To assess the quality of the SPFD model, we computed Bayes factors (BFs) comparing the model with a “null” model (based on the physical frequency differences f2–f1) while accounting for differences in model complexities (e.g., Kass and Raftery, 1995).

### EEG Recording and Analysis

EEG (ActiveTwo; BioSemi) was recorded at 2048 Hz (offline down-sampled to 512 Hz) from 64 electrodes positioned in an elastic cap according to the extended 10–20 system. Individual electrode locations for each participant were obtained prior to the experiment using a stereotactic electrode-positioning system (Zebris Medical GmbH, Isny, Germany). Four additional electrodes were used to register the horizontal and vertical electrooculogram (hEOG and vEOG). For preprocessing, EEG data were first re-referenced to a common average montage, and then high- and low-pass filtered (with cut-off frequencies of 0.5 and 48 Hz, respectively). Eye blink artefacts in the EEG data were corrected using adaptive spatial filtering based on individual calibration data informed by the vEOG signal (see Ille et al., 2002). The artefact-free EEG data were segmented into epochs from −2500 to 1000 ms relative to the time of saccade onset (based on the hEOG signal) in order to examine EEG oscillations before choices were reported (i.e., response-locked analysis). Based on visual inspection, noisy trials were excluded from further investigations (10.5% of trials on average). To get a time-resolved representation of spectral power in the EEG signal, Morlet wavelet transforms of short segments of EEG data were computed every 50 ms. The lengths of these segments depended on the frequency of the applied wavelet (i.e., 4–48 Hz resolved with 1 Hz), and always spanned seven cycles (e.g., 700 ms for 10 Hz, 350 ms for 20 Hz). The resulting TF representations of the EEG data were hence resolved at 50 ms and 1 Hz (i.e., TF bin = 50 ms × 1 Hz). All analyses were done in MATLAB (The MathWorks) using the SPM12 toolbox (Wellcome Department of Cognitive Neurology, London^1^), including the FieldTrip toolbox for EEG/MEG data (Radboud University Nijmegen, Donders Institute^2^).

### Statistical Analysis

The response-locked single-trial TF data were square root transformed (yielding spectral amplitudes) to approximate normally distributed data (see Kiebel et al., 2005). Additionally, TF data were smoothed with a 3 Hz × 300 ms FWHM (full width at half maximum) Gaussian kernel to decrease inter-subject variability (e.g., Kilner et al., 2005; Litvak et al., 2011). For each participant, we used the smooth TF images of all trials to estimate the average TF maps for either choice category (i.e., f2 < f1 and f2 > f1 trials) separately for correct and incorrect decisions. That is, we implemented a general linear model (GLM) with 2x2 factorial design (factors: “f2 < f1/f2 > f1”; “correct/incorrect”), and estimated the interaction terms. We contrasted the average TF maps within each participant to identify interaction effects between both factors (i.e., between “f2 < f1/f2 > f1” and “correct/incorrect”; contrast vector = [−1 1 1 −1]), as this resulted in contrasting the actual choices of participants disregarding whether choices were correct or incorrect (i.e., chose “f2 > f1” vs. chose “f2 < f1”). The resulting contrast images hence showed the difference in spectral amplitude for each TF bin between both choices (i.e., “f2 > f1” choices minus “f2 < f1” choices) considering correct and incorrect trials. To identify time, frequencies, and channels for which this contrast was consistently different from zero across participants, we used cluster-based permutation testing (Maris and Oostenveld, 2007). We compared the summary statistics of the observed data (one-sample *t*-test across contrast images of all participants in each TF bin) with a distribution of summary statistics obtained from 500 randomly sign-flipped permutations. Consistent with our previous work focusing on strong and focal effects (Herding et al., 2016), a cluster was defined as a group of adjacent TF bins that all exceeded a cluster-defining threshold of *p*_threshold_ < 0.001 (uncorrected). Clusters that exceeded a family-wise error (FWE) corrected threshold of *p*_cluster_ < 0.05 (corrected for time, frequency, and channels) were considered to be statistically significant. Additionally, we probed whether a significant modulation by choice was observed individually for correct and incorrect trials within the identified TF cluster. Hence, we computed a conjunction analysis of the choice modulation between correct and incorrect trials (i.e., conjunction of contrasts: [−1 1 0 0] AND [0 0 1 −1]; cf. Friston et al., 2005; Nichols et al., 2005). As described above, we identified significant TF clusters using cluster-based permutation testing separately for correct and incorrect trials, and inspected whether the resulting clusters overlapped. For this analysis, we used a cluster-defining threshold of *p*_threshold_ = 0.01 (uncorrected), and only corrected for channels that displayed a choice-modulation in the previous analysis of interaction effects.

### Source Reconstruction

The cortical sources of choice-modulated beta band activity observed on the scalp-level were localized using the 3D source reconstruction routines provided by SPM12 (Friston et al., 2006). Based on the individually recorded electrode positions for each participant, a forward model was constructed using an 8196-point cortical mesh of distributed dipoles perpendicular to the cortical surface of a template brain (cf. Friston et al., 2006). The lead field of the forward model was computed using the three-shell Boundary Elements Method (BEM) EEG head model available in SPM12. The forward model was inverted using a smoothness prior (called ‘COH’ in SPM; cf. Litvak et al., 2011), which is similar to the LORETA approach (Pascual-Marqui et al., 1994). That is, the inverse solution preferred source activity with only proximal sources showing correlated activity while the total energy of source activity was minimized. Additionally, we applied group constraints for the model inversion, which effectively restricted the inverse solution to explain individual data using the same set of sources across participants (cf. Litvak and Friston, 2008). Preprocessed response-locked single-trial EEG data before TF transformation (i.e., in the time-domain) were used to invert the forward model. Before model inversion, the single-trial data were additionally tailored to the time interval of the choice modulation identified on the scalp level (i.e., −750 to −450 ms before responses were given). According to the interaction terms of the 2x2 factorial design (see above), the results of the model inversion were summarized in four 3D images that reflected average spectral source power in a representative TF window (i.e., 24–32 Hz; −700 to −500 ms from saccade onset). These images were obtained by computing wavelet transforms of single-trial source activity, and then averaging the source power across trials for each condition of interest. The 3D images were then used to contrast source power between choices for each participant, analogously to the conjunction analysis in sensor space (i.e., conjunction of contrasts: [−1 1 0 0] AND [1 −1 0 0]). The conjunction analysis yielded only sources that exhibited significantly higher beta band power for “f2 > f1” choices than for “f2 < f1” choices in both correct and incorrect trials (i.e., testing the conjunction null; cf. Friston et al., 2005; Nichols et al., 2005). The results of this mass-univariate statistical test are displayed at a significance level of *p* < 0.001 (uncorrected) indicating the most probable sources of the effect observed at the sensor-level. Anatomical reference for source estimates was established on the basis of the SPM anatomy toolbox (Eickhoff et al., 2005) where possible.

### Time Courses

To get further insights into the effects obtained from the TF analysis, we extracted underlying time courses from the statistically significant TF cluster separately for correct and incorrect trials. For correct trials, we computed the time courses individually for different levels of SPFDs. Based on all observed SPFD values (differences of log-transformed frequency values), we defined six levels of SPFD (i.e., [< −0.18]; [−0.18 to −0.09]; [−0.09 to 0]; [0 to 0.09]; [0.09 to 0.17]; [> 0.17]). We specified the levels symmetrically around a SPFD of zero (corresponding to chance-level performance), and in such a way that each participant had at least one stimulus pair for each level. Based on the identified TF cluster, we computed the grand average time courses of upper beta band amplitude (24–32 Hz) for each level of SPFD. For incorrect trials, we separated the trials only into two classes (due to low trial numbers for some levels of SPFD) with SPFD < 0 and SPFD > 0, i.e., f2 < f1 and f2 > f1, and computed the grand average time courses.

## Results

### Behavioral Results

On average, participants made correct choices on 74.4% of all stimulus pairs. We performed a within-subject analysis of variance (ANOVA) with the factors “difficulty” (±4 vs. ±2 Hz stimulus differences) and “sign” (positive vs. negative stimulus differences) on proportions of correct responses (PCRs), using a logit-transform to account for non-normality of the residuals. The analysis revealed significant main effects of the factors difficulty (*p* < 0.001) and sign (*p* = 0.001), and a significant interaction of the two factors (*p* < 0.001). As expected, a larger proportion of trials were judged correctly when the physical f2–f1 frequency difference was ±4 Hz (80.9% correct) compared with trials where the difference was only ±2 Hz (67.8%; *p* < 0.001; paired *t*-test; see difficulty effect **Table 1**). We also observed more correct responses for positive (78.1% correct) compared with negative frequency differences (70.6%; *p* = 0.006 paired *t*-test; see sign effect **Table 1**), which indicates an overall response bias toward “f2 > f1” choices (mean criterion shift: 0.12; *p* = 0.003; one-sample *t*-test).

**Table 1:** Behavioral data.

	Frequency difference of stimuli (f2–f1) in Hz					
	−4	−2	2	4	Difficulty effect	Sign effect
PCR (%)	75.9 ± 4.4	65.3 ± 3.5	70.5 ± 4.3	86.1 ± 3.7	n/a (*p* < 0.001)^∗^	n/a (*p* = 0.002)^∗^
RT correct (ms)	590.2 ± 44.8	608.0 ± 48.3	554.5 ± 47.1	541.6 ± 44.7	−15.4 ± 9.0 (*p* = 0.002)^∗^	−51.1 ± 28.6 (*p* = 0.001)^∗^
RT incorrect (ms)	615.9 ± 64.9	593.9 ± 60.7	651.5 ± 58.1	678.6 ± 68.2	24.5 ± 19.0 (*p* = 0.014)^∗^	60.2 ± 34.8 (*p* = 0.002)^∗^

*Proportion of correct responses (PCRs) and response times (RTs) as a function of the physical frequency difference f2–f1. Mean values ± 95% confidence interval (CI) are shown. ‘Difficulty effect’ compares easy (±4 Hz) and difficult (±2 Hz) trials in a paired t-test. ‘Sign effect’ compares between trials with positive (2 and 4 Hz) and negative (−2 and −4 Hz) frequency differences in a paired t-test. PCRs and RTs showed significant effects of difficulty and sign. RTs showed both effects for correct and incorrect trials, however, in opposing directions (cf. interactions in ANOVA of RTs). PCRs were logit-transformed before testing, due to non-normally distributed residuals. We omitted average differences of logit-transformed PCRs for both effects to avoid confusion (indicated by n/a). Asterisks indicate statistically significant results.*

An ANOVA (2x2x2 repeated measures design with factors “correct/incorrect,” “difficulty,” and “sign”) of the median response times (RTs) showed a significant main effect for the factor “correct/incorrect” (*p* < 0.001), and two significant interactions (“correct/incorrect” × “sign”, *p* = 0.001 and “correct/incorrect” × “difficulty”, *p* = 0.004). More precisely, the median RT with respect to f2 stimulus onset was on average shorter for correct trials (570.4 ms) than for incorrect trials (620.5 ms; *p* < 0.001; paired *t*-test). For correct trials, RTs were faster for trials with f2 > f1 (548.1 ms) as compared to f2 < f1 (599.1 ms; *p* = 0.001; paired *t*-test), whereas for incorrect trials the pattern was reversed (665.1 ms when f2 > f1, and 604.9 ms when f2 < f1; *p* = 0.002; paired *t*-test; all patterns of interaction effects in the RT data are detailed in **Table 1**). Thus, participants were in general faster when choosing “f2 > f1,” no matter whether this choice was correct or incorrect. This is in line with the overall response bias toward “f2 > f1” choices (see above). Accordingly, when computing criterion shifts separately for fast and slow trials of each participant (i.e., median split of RTs), fast responses displayed a much stronger bias toward “f2 > f1” choices than slow responses (*p* < 0.001, paired *t*-test). In fact, whereas participants clearly favored “f2 > f1” choices in fast trials (mean criterion shift: 0.31; *p* < 0.001, one sample *t*-test), in slow trials the bias was actually reversed (mean criterion shift: −0.11; *p* = 0.009, one sample *t*-test).

### Upper Beta Band Oscillations in Right Frontal Electrodes Encode Choices before Responding

**FIGURE 2:**
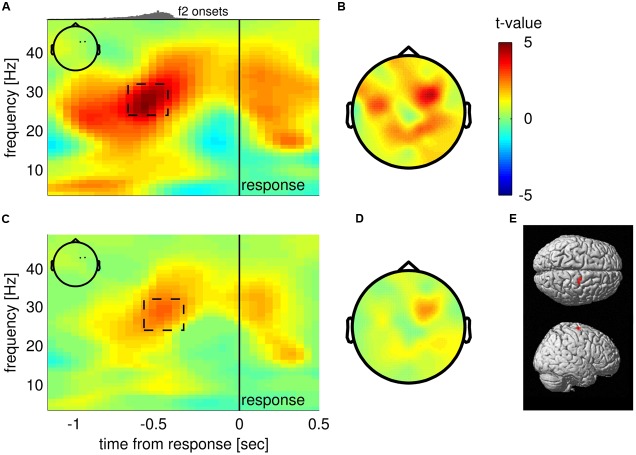
**Choice-selective modulation of upper beta band amplitude. (A)** Time-frequency (TF) map displaying *t*-values from group analysis of interaction effect (“f2 < f1/f2 > f1” × “correct/incorrect”), averaged over electrodes FC2 and FC4 (see inset) spanning a statistically significant cluster. Histogram on top indicates the distribution of stimulus onset times of the second stimulus. **(B)** Scalp topography of TF window centered on significant cluster as indicated in **(A)**. **(C)** Results of the conjunction analysis between correct and incorrect trials averaged over electrodes FC2 and FC4 (inset). The TF map displays the minimum of *t*-values when combining choice-selective modulation computed separately for correct and incorrect trials. **(D)** Scalp topography corresponding to the TF window indicated in **(C)**. **(E)** Most likely source location of the choice-selective beta band modulation.

To test if choices were reflected in oscillatory EEG activity before a response was given, we compared average TF maps of f2 < f1 and f2 > f1 trials in response-locked data, while considering that any possible effect of choice should switch sign between correct and incorrect trials (i.e, we checked for an interaction effect of the factors “f2 < f1/f2 > f1” and “correct/incorrect”). The analysis revealed that upper beta band amplitude (∼24–32 Hz) in right frontal electrodes (FC2, FC4; inset **Figure 2A**) was significantly higher for “f2 > f1” choices well before responses were given (−750 to −450 ms from response; *p*_cluster_ = 0.034, FWE corrected; **Figure 2A**, dashed rectangle). The scalp topography of the TF cluster shows that the effect also spreads to parietal electrodes and displays a second, weaker peak in left frontal electrodes (**Figure 2B**; the cluster extended to both sites for a lower cluster-defining threshold of *p*_threshold_ = 0.01). Notably, steady-state evoked potentials (SSEPs) of vibrotactile stimuli are known to lead to a narrow-band power increase in the EEG signal at frequencies corresponding to the stimulus frequency in electrodes contralateral to stimulation (e.g., Tobimatsu et al., 1999). For f2 > f1 trials, f2 was generally higher (25 Hz on average) than for f2 < f1 trials (19 Hz on average). Hence, correct choices of “f2 > f1” were primarily accompanied by SSEPs in the upper beta band, whereas correct choices of “f2 < f1” were mainly associated with SSEPs in lower frequencies. Given that the reported effect partly overlapped with the presentation of f2, we were concerned whether the alleged choice-selective modulation of upper beta band amplitude was driven by the systematic differences in SSEPs between choices. Importantly however, the systematic relationship between SSEPs and choices can only compromise our findings for correct trials. Therefore, we computed a conjunction analysis between correct and incorrect trials to probe whether the observed beta band modulation was the same for both correct and incorrect trials. Indeed, we found overlapping significant TF clusters in the upper beta band (∼25–30 Hz) approximately 500 ms before responses were given in previously identified electrodes FC2 and FC4 (correct: −600 to −400 ms; 26–35 Hz; *p*_cluster_ = 0.044; incorrect: −1000 to −400 ms; 20–33 Hz; *p*_cluster_ = 0.004; cf. **Figure 2C**). Remarkably, the effect was even stronger for incorrect trials than for correct trials. Displaying the minimum *t* statistics between correct and incorrect trials reveals that only right frontal electrodes show the choice-selective modulation of upper beta band amplitude consistently for correct and incorrect trials (**Figures 2C,D**). Accordingly, the most probable source of the effect was found in the right precentral gyrus including FEF (MNI coordinates of cluster peak: 18, −12, 70; *p* < 0.001, uncorrected; **Figure 2E**). Taken together, we can largely rule out a major contribution of SSEPs to the observed beta band modulation.

**FIGURE 3:**
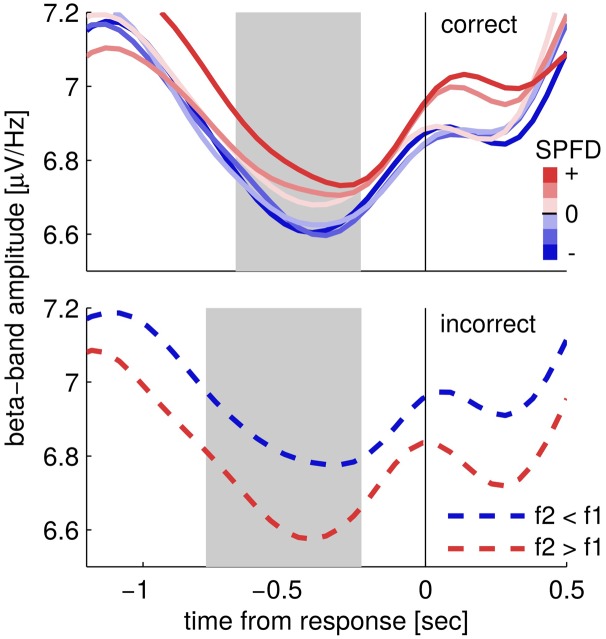
**Time courses of upper beta band amplitude (24–32 Hz) separately for correct (upper) and incorrect trials (lower).** Correct trials are split into six levels of subjectively perceived frequency differences (SPFDs) as inferred from a Bayesian inference model that describes choice behavior in this task better than physical differences (see text for details). Despite this fine-grained partitioning, time courses are separated solely according to choice categories. Incorrect trials were split only into two classes (according to f2 > f1 and f2 < f1, due to low trial numbers) and still showed a higher beta band amplitude for (incorrect) “f2 > f1” choices (i.e., f2 < f1, blue line) than for “f2 < f1” choices (i.e., f2 > f1, red line). Shaded areas denote the time interval in which the second stimulus was typically presented (central 50%).

Next, we looked at the choice-selective beta band modulation independently for correct and incorrect choices by separately computing the according grand mean time courses of upper beta band amplitude (24–32 Hz; **Figure 3**). The time courses for correct trials show that beta band amplitudes separate categorically according to choices (**Figure 3**; correct trials). That is, the according choice category modulated upper beta band amplitude, but not the specific values of the SPFD. Notably, the SPFDs described participants’ choices more accurately than the physical differences in each trial (strong evidence in favor of our model, i.e., BFs > 20, for 20/22 participants). For incorrect trials, we only distinguished between SPFD > 0 and SPFD < 0 (i.e., f2 > f1 and f2 < f1), and found that upper beta band amplitude was still higher for (incorrect) choices of “f2 > f1” (**Figure 3**; incorrect trials). Reiterating the results of our conjunction analysis, the identified modulation of beta band amplitude by choices was neither driven solely by correct trials nor solely by incorrect trials. Interestingly, for incorrect trials beta band amplitude was separated according to choices already well before the presentation of the second stimulus. Such a pre-stimulus difference might possibly explain why participants made erroneous choices in according trials (i.e., as the result of a bias), and would foster the interpretation of upper beta band amplitude as a precursor of the ensuing decision report.

In a control analysis, we examined whether the observed modulation of upper beta band amplitude was possibly related to the present variations in RTs according to choices. In particular, RTs for “f2 > f1” choices were always faster as for “f2 < f1” choices, for both correct and incorrect trials. That is, the same interaction as in the EEG data was also present in RTs (see **Table 1**). Thus, if faster RTs were associated with higher beta band amplitude in electrodes FC2 and FC4, the RT variations would be an alternative explanation of the observed modulation in beta band amplitude. We computed correlations between single-trial RTs and beta band amplitude for each participant, however, the obtained correlation coefficients scattered randomly around zero across participants (one sample *t*-test of correlation coefficients; mean ρ = −0.021, *p* = 0.245). Additionally, we checked for the same correlation within each choice category, but again, did not find any connection (one sample *t*-test of correlation coefficients; “f2 > f1” choices: mean ρ = −0.013, *p* = 0.463; “f2 < f1” choices: mean ρ = −0.018, *p* = 0.408). Hence, we can largely rule out that the reported modulation of beta band amplitude can be attributed to systematic RT variations. We also probed whether the overall response bias toward “f2 > f1” choices could explain the observed modulation in the beta band. To this end, we repeated the main analysis only using data from participants showing no such bias, or even a bias in the opposite direction (criterion shift < 0.1, 10 participants). These participants did also not show systematic differences in RTs between choices (i.e., “f2 > f1” vs. “f2 < f1” choices) neither for correct nor for incorrect trials (paired *t*-test between choices, *p* = 0.224 and *p* = 0.352). Despite the markedly reduced sample size, we observed the same pattern of upper beta band amplitude being higher for “f2 > f1” choices than for “f2 < f1” choices.

**FIGURE 4:**
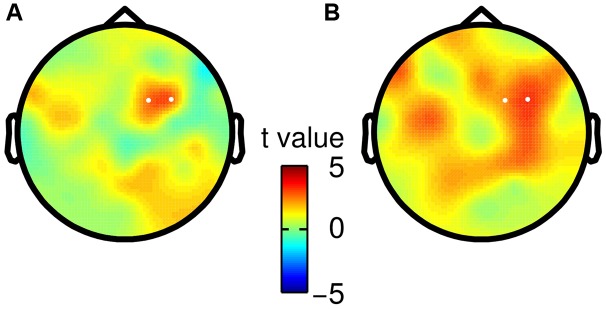
**Scalp topographies of choice-selective beta band modulation for both saccade-to-choice mappings.** White dots correspond to electrodes spanning the significant TF cluster in the main analysis based on all participants. **(A)** Choices of “f2 > f1” were associated with a rightward saccade, whereas “f2 < f1” choices required a leftward saccade. **(B)** Opposite mapping as described in **(A)**.

Finally, we tested whether the observed choice-selective modulation in the beta band was consistent for both specific mappings of choices onto saccade directions. Hence, we split participants according to their response mapping (i.e., right saccade = “f2 > f1” or right saccade = “f2 < f1”), and repeated the analysis of TF data separately for both groups (*N* = 11). We did not find any statistically significant differences between both groups (independent two-sample *t*-test, no clusters with *p* < *p*_threshold_ before saccade onset), but rather found a considerable agreement in the topography of the choice-selective beta band modulation (**Figure 4**).

## Discussion

In the current study we investigated oscillatory EEG signatures of perceptual decisions based on the comparison between two sequentially presented vibrotactile frequencies f1 and f2. Participants decided whether f2 > f1 or f2 < f1 by performing a horizontal saccade, where the association between saccade direction and choice was counterbalanced across participants. We found that the amplitude of upper beta band oscillations (∼24–32 Hz) in right frontal electrodes was modulated by participants’ choices before responses were given, regardless of whether choices were correct or incorrect, and independent of the specific saccade-to-choice mapping. In particular, “f2 > f1” choices were always associated with a higher beta band amplitude than “f2 < f1” choices. Notably, the same modulation pattern of beta band amplitude was recently shown when participants (non-human primates and humans) completed the same task, but reported choices by button presses (Haegens et al., 2011; Herding et al., 2016). In analogy to these studies, we found in the current data that premotor areas were implicated as the most likely source of the choice-selective signal, however, now with a focus on distinct lateral parts, including FEF.

The crucial role of premotor cortex in decision formation during the vibrotactile 2AFC task was established by the seminal work of Romo and colleagues with non-human primates (reviewed in Romo and de Lafuente, 2013). Electrophysiological recordings in mPMC and vPMC showed choice-selective differences in premotor firing rates before actual responses were given by button presses (Hernández et al., 2002, 2010; Romo et al., 2004). Similar to the current data, this modulation was observed as early as during the presentation of the second stimulus (Hernández et al., 2002, 2010; Romo et al., 2004), and was shown to be behaviorally relevant, as the modulation was inverted for incorrect choices (Hernández et al., 2002). Conversely, the choice-selective differences in firing rates disappeared when no comparison of f1 and f2 was necessary in order to respond (i.e., a visual cue guided action), dissociating the finding from mere motor preparation (Hernández et al., 2002, 2010; Romo et al., 2004). To dissociate specific left/right saccade preparation (i.e., lateralized parietal alpha/beta band decrease; see Carl et al., 2016) from choices in the current study, we counterbalanced the mapping from saccade direction to choice across participants. We found that both mappings led to very similar results when according data were analyzed separately (i.e., for either half of the participants). Hence, the reported choice-selective modulation of beta band amplitude is most likely independent of specific saccade preparation. Moreover, we did not find any additional lateralized choice effects (i.e., for neither half of the participants) as a consequence of a consistent mapping between saccade direction and choice (cf. lateralized beta band decrease before decision reports by button presses, e.g., Donner et al., 2009).

Typically, beta band oscillations (∼15–25 Hz) are associated with sensorimotor processing. That is, beta band amplitude is known to decrease over somatosensory areas in anticipation and during the presentation of tactile stimuli, as well as to rebound afterwards (e.g., Jasper and Andrews, 1938; Pfurtscheller, 1981; Bauer et al., 2006; van Ede et al., 2011). In preparation for and during voluntary hand movements like button presses, the same pattern of beta band decrease followed by a rebound over contralateral motor areas is also reliably observed (e.g., Jasper and Penfield, 1949; Pfurtscheller, 1981). Likewise, several studies suggest that a decrease in beta band amplitude over contralateral posterior parietal areas accompanies the execution of saccades (e.g., Pesaran et al., 2002; Brignani et al., 2007; Carl et al., 2016). Moreover, Jo et al. (2016) recently reported a negative correlation between the level of beta band amplitude over motor areas before initiating voluntary button presses and according RTs. Given that in the current study RTs varied systematically in the same way as the (upper) beta band was modulated by choice (i.e., faster responses for “f2 > f1” than for “f2 < f1” choices for correct and incorrect trials), we carefully examined whether the observed beta band modulation could be attributed to these RT variations. However, RTs were not correlated with upper beta band amplitude, neither over all trials, nor within the separate choice categories (i.e., “f2 > f1” or “f2 < f1”). More likely, the variations in RTs are related to the observed response bias toward “f2 > f1” choices, i.e., the preferred choice is also accompanied by faster responses. In favor of this interpretation, fast trials exhibited a stronger bias than slower trials. Moreover, the bias disappears when introducing a response delay to the task (unpublished observation), suggesting that the tendency for choosing “f2 > f1” might be confined to decisions under time pressure. To rule out that the response bias itself accounts for the observed beta band modulation, we additionally analyzed EEG data separately for participants that showed no substantial bias (or even a bias in the opposite direction) and no systematic RT differences between choices. Despite the reduced sample size, we still found the same tendency of “f2 > f1” choices being accompanied by higher beta band amplitude than “f2 < f1” choices, for correct and incorrect trials. Taken together, the reported modulation of upper beta band amplitude by participants’ choices is unlikely to be related to systematic shifts of sensorimotor beta band effects due to RT variations or an overall response bias.

Rather, our finding aligns well with previous work that established a link between prefrontal upper beta band oscillations and WM content in the same task (i.e., f1 values; see Spitzer et al., 2010; Spitzer and Blankenburg, 2011), and thus further supports the notion of upper beta band oscillations encoding different task-relevant entities at according processing stages of the vibrotactile 2AFC task (cf. Herding et al., 2016). In the context of decision making, given location (i.e., premotor areas) and characteristics (i.e., representation of content on which choice is based, independent of specific motor response) of the observed effect, we propose that this entity might reflect the input to the (pre)motor system which is in charge of the subsequent response. In particular, beta band amplitude might signal the decision outcome which in turn informs the ensuing action that is planned in effector-specific brain areas. How the beta band modulation might be implemented in detail, however, remains an open question. A recently proposed biophysically principled computational model was able to reproduce beta bursts in human MEG and animal LFPs (monkey and mouse) in great detail (Sherman et al., 2016). Interestingly, the model predicts modulations of the burst amplitudes by changes in the firing rates of some neurons in the network. Hence, this model might provide a new angle on how the firing rate code revealed by Romo and colleagues (e.g., see Romo and de Lafuente, 2013 for review) might be directly translated into amplitude modulations in the beta band as reported here, and in previous work (Haegens et al., 2011; Herding et al., 2016).

Besides the considerable agreement between our current results and previous work in the vibrotactile 2AFC task, the findings presented here are notably the first ones based on decisions with saccade responses in this paradigm. In the visual domain, however, extensive research has investigated perceptual decision making utilizing saccades for responding in non-human primates (reviewed in Glimcher, 2003; Gold and Shadlen, 2007). The large body of work compiled by Shadlen and colleagues presents coherent evidence that choices, which are expressed by saccades, are reflected in the firing rates of various oculomotor brain areas, i.e., LIP (e.g., Shadlen and Newsome, 1996), FEF (e.g., Hanes and Schall, 1996; Kim and Shadlen, 1999), and SC (e.g., Ratcliff et al., 2003). More precisely, in the random dot motion (RDM) task, LIP activity was shown to reflect the accumulated evidence (i.e., motion information) provided by visual area MT (e.g., Ditterich et al., 2003; Hanks et al., 2006) peaking at RT (e.g., Shadlen and Newsome, 2001). A similar accumulation-to-bound signal was found in FEF (Hanes and Schall, 1996) and SC (Ratcliff et al., 2003) using a visual search task. In general, LIP, FEF, and SC seem to play similar roles in saccade target selection and spatial attention by implementing salience or relevance maps with gradually less abstract representations of the visual field (see e.g., Colby and Goldberg, 1999; Andersen and Buneo, 2002; Fecteau and Munoz, 2006; Schall, 2015). In the visual RDM task, however, Katz et al. (2016) recently questioned the causal role of LIP for decision making by showing that a pharmacological inactivation had no effect on task performance, whereas area MT (i.e., the momentary evidence) proved to be indispensable. Notably, the source reconstruction of the present choice-selective modulation of upper beta band modulation suggested areas in the precentral gyrus including FEF as likely sources. Hence, our findings are remarkably consistent with the work in non-human primates investigating decisions reported by saccades (cf. Hanes and Schall, 1996; Kim and Shadlen, 1999). Contrasting the results from the current study with our previous work, in which participants completed the same task but responded with button presses, reveals that the signal (i.e., choice-selective modulation of upper beta band amplitude) remained the same, however, the topography and the suggested source locations differ considerably. In particular, whereas button press responses implied medial premotor areas as a putative source of the choice signal, saccade responses hinted at source locations including FEF. Hence, both studies observed the same choice-selective signal, however, found sources that are associated with the planning of respective motor responses in an effector specific way.

In line with the aforementioned studies, our findings thus support the notion of an intentional framework of decision making (e.g., Cisek, 2007; Shadlen et al., 2008; Cisek and Kalaska, 2010), which proposes that decisions are expressed in form of intentions to act. As a consequence, neural correlates of decision making should be found in brain areas that are involved in the planning/preparation of the action that is used to express the choice, independent of the specific task at hand. In this light, also the work of Romo and colleagues is in agreement with an intentional framework of decision making. Choices in the vibrotactile 2AFC task were always reported by button presses, and choice-selective neuronal activity was found in mPMC and vPMC (Hernández et al., 2002, 2010; Romo et al., 2004; Haegens et al., 2011). Here, we provide novel evidence that a combination of the vibrotactile 2AFC task with another response modality (i.e., saccades) translates the choice-selective signal to corresponding effector-specific brain areas. Hence, we could effectively bridge the gap between the work of Romo and colleagues (vibrotactile 2AFC) and the work of Shadlen and colleagues (oculomotor responses), and show that their findings are transferable within an intentional framework of decision making.

## Author Contributions

JH, SL, and FB designed the study. JH and SL collected the data. JH, SL, and FB analyzed the data, interpreted the results, and wrote the manuscript. All authors approved the final version of the manuscript for submission.

## Conflict of Interest Statement

The authors declare that the research was conducted in the absence of any commercial or financial relationships that could be construed as a potential conflict of interest.

## Footnotes

1 http://www.fil.ion.ucl.ac.uk/spm

2 http://www.fieldtriptoolbox.org
